# Hepatoprotective and antioxidant activities of extracts from Salvia–Nelumbinis naturalis against nonalcoholic steatohepatitis induced by methionine- and choline-deficient diet in mice

**DOI:** 10.1186/s12967-014-0315-x

**Published:** 2014-11-19

**Authors:** Yang Liu, Haiyan Song, Lei Wang, Hanchen Xu, Xiangbing Shu, Li Zhang, Ying Li, Dongfei Li, Guang Ji

**Affiliations:** Institute of Digestive Diseases, Longhua Hospital, Shanghai University of Traditional Chinese Medicine, Shanghai, 200032 China; Department of Liver Diseases, Longhua Hospital, Shanghai University of Traditional Chinese Medicine, Shanghai, 200032 China; Department of Traditional Chinese Medicine, East Hospital, Tongji University, Shanghai, 200120 China

**Keywords:** Salvia–Nelumbinis naturalis, NASH, Liver injury, Oxidative stress

## Abstract

**Background:**

Nonalcoholic steatohepatitis (NASH), the advanced stage of nonalcoholic fatty liver disease that is characterized by both steatosis and severe injury in liver, still lacks efficient treatment. The traditional Chinese formula Salvia–Nelumbinis naturalis (SNN) is effectively applied to improve the symptoms of nonalcoholic simple fatty liver (NAFL) patients. Previous studies have confirmed that SNN could reduce the liver lipid deposition and serum transaminases of NAFL experimental models. This study aims to determine whether SNN is effective for murine NASH model and investigate the underlying pharmacological mechanisms.

**Methods:**

C57BL/6 J mice were fed with methionine- and choline-deficient (MCD) diet for six weeks to induce NASH. Simultaneously, SNN or saline was intragastrically administered daily to the mice in the SNN or model group, respectively. A standard diet was given to the control mice. Serum biochemical indices and tumor necrosis factor-α were measured. Liver histopathology was observed, and the contents of triglycerides and lipid peroxide malondialdehyde (MDA) in liver homogenates were evaluated. The hepatic expression and/or activation of genes associated with inflammation, apoptosis, and oxidative stress were determined by quantitative RT-PCR or Western blot analysis.

**Results:**

The prominent liver steatosis displayed in the NASH model was prevented by SNN. The liver injury of NASH mice was obviously manifested by the increased levels of serum transaminases and bilirubin, as well as the lobular inflammation, elevated pro-inflammatory cytokines, and upregulated apoptosis in liver tissues. SNN administration improved the aforementioned pathological changes. The increased hepatic levels of MDA and cytochrome P450 2E1 of the model confirmed the unregulated balance of oxidative stress. The hepatic expression of nuclear factor erythroid 2-related factor 2 and its target genes decreased, whereas c-Jun N-terminal kinase activation in the model mice increased. Treating the mice with SNN significantly improved oxidative stress-related harmful factors.

**Conclusions:**

This study shows that SNN can protect the liver from severe steatosis and damage induced by MCD diet, which suggests the potential use of SNN on the treatment of NASH patient. The results also indicate that improving the hepatic antioxidant capability of the liver may contribute to the underlying hepatoprotective mechanism.

## Background

Nonalcoholic fatty liver disease (NAFLD) is an increasing prevalent health problem that ranges from simple fatty infiltration of the liver parenchyma (nonalcoholic simple fatty liver, NAFL) to steatosis with inflammation and hepatocellular ballooning (nonalcoholic steatohepatitis, NASH) and ultimately cirrhosis [1]. Patients with NAFL appear to have a nonprogressive course with benign prognosis. However, approximately 10% to 30% of NAFL patients develop NASH with more serious form of liver damage, which may further progress to cirrhosis in as many as 25% of the cases and suffer from its complications, including portal hypertension, liver failure and hepatocellular carcinoma [2].

NAFLD begins with an aberrant lipid accumulation in the liver [3], followed by initial changes that induce the liver to be sensitive to oxidative stress (OS) and pro-inflammatory cytokines [4]. OS is an etiological factor in many acute and chronic liver diseases and plays a critical role in the progression of NAFL to NASH [5]. Sources of OS include elevated level of cytochrome P450 2E1 (CYP2E1), lipid peroxidation, and cytokine induction [6,7]. CYP2E1 is an inducible enzyme hydroxylating fatty acid that can initiate lipid peroxidation process [8,9]. CYP2E1 is frequently induced in NASH, leading to increased reactive oxygen species (ROS) that trigger OS [8]. Considerable OS causes lipid peroxidation of cell membrane and activation of hepatic stellate cell, resulting in inflammation, apoptosis, and fibrogenesis [10]. Increased OS can enhance the secretion of inflammatory cytokines and hepatocyte apoptosis, which possess pivotal roles in the pathogenesis of liver damage in NASH [11-13]. Pro-inflammatory cytokines, such as tumor necrosis factor-α (TNF-α) and interleukin-1β (IL-1β), which are involved in liver injury and repair, are also considered as NASH indicators [14-17]. TNF-α plays an important role throughout the progression of steatosis to NASH [12]. The effects of TNF-α result in additional lipid peroxidation of mitochondrial membranes, thereby further enhancing OS in NASH [18]. Therefore, inhibiting TNF-α can suppress liver injury in NASH [19].

Valuable therapeutic interventions against NASH remain limited; hence, searching for a safe and effective drug is relatively essential [20]. Salvia–Nelumbinis naturalis (SNN) formula (initially called Jiang Zhi Granule) is a compound prescription in traditional Chinese medicine composed of five medicinal herbs, namely, *Salviae* (Danshen in China), *Nelumbinis* (Heye in China), *Herba Gynostemmatis* (Jiaogulan in China), *Rhizoma Polygoni Cuspidati* ( Huzhang in China), and *Herba Artemisiae scopariae* (Yinchen in China) [21,22]. This complex prescription has been used to treat NAFL in clinical practice in China, with significant effects of alleviating hepatic steatosis with few side effects [23]. In 2008, SNN was granted the permission and certification by the Chinese SFDA (No. 2008 L11181) to be used as drug for clinical trials. SNN can improve the liver to spleen ratio (L/S ratio) and decrease the body mass index of NAFL patients [23]. Previous *in vivo* studies have also confirmed the effect of SNN on NAFL and partially unraveled its underlying mechanisms, including improving leptin and insulin resistance, inhibiting transcription of liver X receptor α (LXR-α)-mediated sterol regulatory element binding protein-1c (SREBP-1c), and maturation of SREBP-1c independent of LXR-α [24-27]. *In vitro* studies have indicated that the components extracted from SNN reduce lipid droplet deposition and increase the resistance to damage of hepatocytes induced by free fatty acids [21,28]. Taken together, our previous studies mainly focused on the therapeutic function of SNN on simple steatosis. Therefore, in the present study, we tried to determine whether and how SNN is potentially effective for NASH. For this objective, the NASH model of mouse induced by methionine- and choline-deficient (MCD) diet was used, of which the major pathological factors are excess OS and failure of lipid transport from hepatocytes [29]. SNN extract was administered to the NASH model to observe the efficacy and to further investigate the related molecular mechanism of the traditional formula.

## Methods

### Experimental animals

Male C57BL/6 J mice weighing 20 g to 25 g were purchased from Shanghai SLAC Laboratory Animal Technology Company (License No. SCXK (HU) 2007–0005). The animals were housed in a standard 12 h light/dark cycle at 22 ± 2°C with 55 ± 10% humidity and had access to food and water. The animal experiments were approved by the Institutional Animal Care and Use Committee of Shanghai University of Traditional Chinese Medicine. All animal procedures were performed in accordance to the guide for the care and use of laboratory animals [30].

### Drug and chemicals

SNN was provided by the Department of Pharmacy of Longhua Hospital, Shanghai, China. This herbal formula was triturated and blended to powder. Subsequently, 20 mg of the powder was extracted with 25 mL of water/methanol (5:95, V/V) in an ultrasonic bath at 42 kHz frequency for 30 min. The chemical profile of SNN has already been analyzed by ultra-performance liquid chromatography [28]. SNN was stored at 4°C and diluted to the desired concentrations in distilled water at the time of administration. Hematoxylin–eosin (HE) solution was purchased from Yixin Biological Technology, Inc. (Shanghai, China), and Oil red O was from Sigma-Aldrich (St. Louis, MO, USA). All reagents were at least of analytic grade and applied according to manufacturer’s instructions.

### Experimental design of animals

A total of 36 C57BL/6 J mice were adaptively fed for 7 days and were randomly allocated into the following experimental groups (n = 12 per group) according to their body weights: (1) the control mice given a standard control diet, (2) the model mice fed with MCD diet, and (3) mice fed with MCD diet together with oral garvage of SNN (860 mg/kg body weight) once a day. The application dose of SNN was calculated based on the standard dose in clinical practice, previous *in vivo* experiments, dose conversion among animals and human, and extract yield [22,25,26,28]. The MCD and control diets were purchased from Research Diets, Inc. (New Brunswick, NJ, USA). At the end of the sixth week, all animals were fasted overnight and sacrificed. The serum was separated for further investigation. Mice livers were weighed and frozen or fixed in 10% formalin.

### Serum biochemical analysis

The alanine aminotransferase activity (ALT), aspartate aminotransferase activity (AST), total bilirubin (TBIL), total cholesterol (TC), triglycerides (TG), high-density lipoprotein cholesterol (HDL-c), and low-density lipoprotein cholesterol (LDL-c) were determined to assess liver function using commercially available kits (Jiancheng Institute of Bio Engineering, Inc., Nanjing, China) according to the manufacturer’s instructions. The serum concentration of TNF-α was measured by a Bio-Plex assay kit (Bio-Rad, Hercules, USA) with the Bio-Plex 200 system (Bio-Rad).

### Histological examination

The liver sections were stained with HE according to the standard methods. In brief, the fresh liver tissue samples were fixed in 10% formalin and embedded in paraffin. The samples were cross-cut into slices of 4 μm to 5 μm and stained with HE staining solution. Finally, the stained sections were observed and photographed under a light microscope (with 200× magnification). All HE stained sections (n = 12) were evaluated in a blinded manner by two pathologists for NAFLD activity score (NAS) including the components of steatosis, inflammation and hepatocyte ballooning. The scoring system was as follows: steatosis grade (0–3; 0: <5%, 1: 5%–33%, 2: 33%–66%, and 3: >66%), lobular inflammation (0–3; 0: no foci, 1: few foci/200×, 2: many foci/200×, and 3: >4 foci/200× scope), and balooning (0–2; 0: no foci, 1: <2 foci/200×, and 2: 2–4 foci/200× [31].

### Oil Red O staining

For Oil Red O staining, a stock solution of Oil Red O (0.5 g/100 mL) in isopropanol was prepared, and protected from light. After being fixed with 10% paraformaldehyde for 30 min, the frozen liver sections with 10 μm thickness were stained with Oil Red O for 60 min. Nonspecific staining was removed with 70% ethanol. The sections were counterstained with hematoxylin diluted at 1:10 and mounted with 80% glycerol. The stained sections were visualized and photographed under a microscope (Olympus IX71, Tokyo, Japan).

### Evaluation of lipid content in liver tissues

Exactly 3 ml of Ethanol–acetone (1:1) was added to the hepatic tissues (200 mg), which was then homogenized in an ice bath and mixed thoroughly at 4°C overnight. After 24 h, the liver tissues were centrifuged at 3000 rpm and 4°C for 20 min. Subsequently, the supernatant was transferred to a new tube, and TG was measured based on the instructions on TG assay kit (Jiancheng Institute of Bio Engineering, Inc.) using the colorimetric method.

### Measurement of MDA in liver tissues

The contents of malondialdehyde (MDA) in the liver tissues were determined through thiobarbutaric acid (TBA) method using a MDA testing kit (Beyotime Institute of Biotechnology, shanghai, China). Briefly, the liver tissues (200 mg) were homogenized in 0.15 M KCl solution. The homogenate and 10% trichloroacetic acid were mixed (1:1) and centrifuged. The supernatant was suspended into TBA. After placing in a water bath for 15 min, the samples were centrifuged at 1000 g for 15 min. The optical density of the supernatants was measured at 532 nm with a BioTek SynergyH4 enzyme-labeled instrument (USA).

### Quantitative reverse transcription polymerase chain reaction (RT-PCR)

The changes of different genes in the liver tissues of MCD mice were validated by conducting quantitative RT-PCR. The total RNA was converted to cDNA by using reverse transcription kits (Promega, Madison, WI, USA). All primers were synthesized by Shanghai Shine Gene Company. The sequences of the primers used in this study are indicated in Table 1. Quantitative RT-PCR was then performed using a SYBR Green PCR Master Mix kit (Applied Biosystems, Carlsbad, CA, USA) according to the manufacturer’s protocol. Amplification of β-actin was performed in parallel as a relatively invariant internal reference. The 2^−ΔΔCt^ method was applied for data analysis.

**Table 1:** **The primer sequences for quantitative RT- PCR used in this study**

**Gene**	**Primer sequence**
β-actin	Forward : 5′-GAGACCTTCAACACCCCAGC-3′
	Reverse : 5′-ATGTCACGCACGATTTCCC-3′
TNF-α	Forward : 5′-CCCTCCAGAAAAGACACCATG-3′
	Reverse : 5′-CACCCCGAAGTTCAGTAGACAG-3′
IL-1β	Forward : 5′-TCGTGCTGTCGGACCCAT-3′
	Reverse : 5′-GGCTTGTGCTCTGCTTGTGA-3′
CYP2E1	Forward : 5′-AGGCTGTCAAGGAGGTGCTAC-3′
	Reverse : 5′-GTTTCCCCATTCCCCAGTC-3′
Nrf2	Forward : 5′-TCTTCCATTTACGGAGACCCA-3′
	Reverse : 5′-GATTCACGCATAGGAGCACTG-3′
Nqo1	Forward : 5′-TCAACTGGTTTACAGCATTGGC-3′
	Reverse : 5′-GCTTGGAGCAAAATAGAGTGGG-3′
Gstp1	Forward : 5′-ACCCTGCTGTCCCAGAACC-3′
	Reverse : 5′-GCGAGCCACATAGGCAGAG-3′

### Western blot

Protein concentration was determined using a BCA protein assay kit (CoWin Bioscience, Beijing, China). The protein sample was resolved by 10% denaturing sodium dodecyl sulfate polyacrylamide gel electrophoresis and was blotted onto Immobilon-P PVDF membranes (Millipore, Billerica, MA, USA). After being blocked by 5% milk, the membrane was incubated with primary antibodies at 4°C overnight. The antibodies against c-Jun N-terminal kinase (JNK), Phospho-JNK, activated Caspase-3, Bcl-2-associated X (Bax) protein, and Bcl-2 were purchased from Cell Signaling Technology (Danvers, MA, USA). The antibody against CYP2E1 was obtained from Abcam (Cambridge, MA, USA), and β-actin was obtained from Huaan Biological Technology (Hangzhou, China). Subsequently, the membrane was incubated in a horseradish peroxidase-conjugated goat anti-rabbit or anti-mouse secondary antibody from Thermo Scientific (Rockford, IL, USA) for 1 h. The signal was visualized by enhanced chemiluminescence HRP substrate (Millipore, Billerica, MA, USA) and acquired by GBOX Chemi XT4 System (Syngene, Cambridge, UK). GeneTools software (Syngene) was used for quantification.

### Statistical analysis

The data were expressed as mean ± standard deviation. Statistical analyses were carried out using one-way ANOVA, followed by Tukey’s post-hoc test to assess the differences between the two groups. The data from qPCR, Western blot and pathological scores were compared using Kruskal–Wallis ANOVA test followed by Dunn’s multiple comparison test. SPSS 18.0 was used for all statistical analyses. *P* <0.05 was considered statistically significant.

## Results

### SNN reversed the changes in body and liver weights and serum lipid of the NASH model

**Figure 1:**
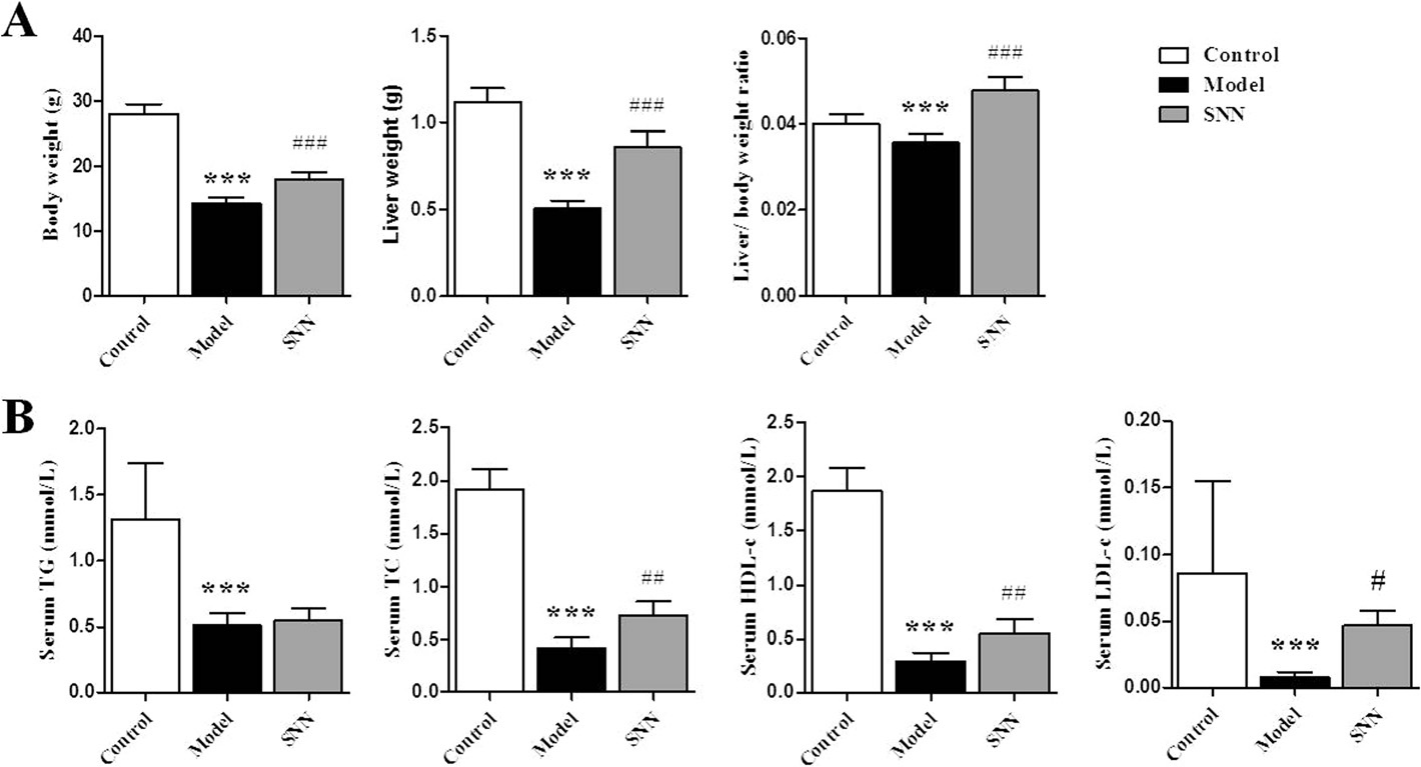
**Changes in body and liver weight and serum lipid of mice. (A)** Body weight, liver weight, and liver-body weight ratio (n = 12 per group). **(B)** Serum TG, TC, HDL-c, and LDL-c (n = 8 per group). Data are expressed as mean ± SD, ***P < 0.001 *vs.* control; ^*###*^ *P* < 0.001, ^*##*^ *P* < 0.01 *vs.* model.

The body and liver weights of mice were measured at the end of the experiment. In contrast to those of the control group, the body weight, liver weight, and liver–body weight ratio remarkably decreased in the NASH model (*P* <0.001). These parameters significantly improved in mice treated with SNN (*P* <0.001) (Figure 1A).

Statistically significant differences were also observed in the blood lipid content. The serum levels of TG, TC, HDL-c, and LDL-c decreased in the model group (*P* <0.001), which were prevented by SNN intervention to a certain extent, particularly the levels of TC, HDL-c (*P* <0.01), and LDL-c (*P* <0.05) (Figure 1B).

### SNN ameliorated hepatosteatosis induced by MCD diet

**Figure 2:**
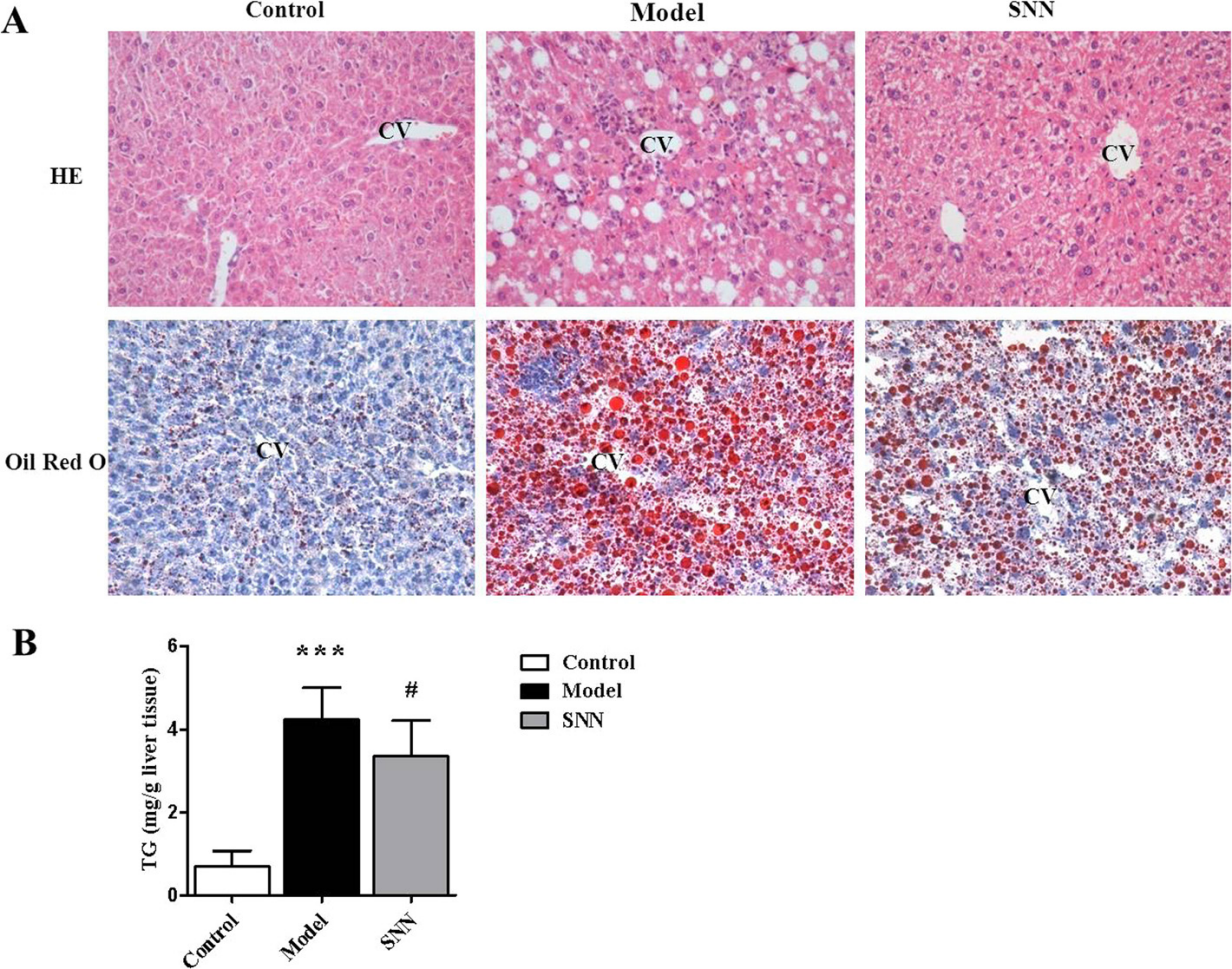
**Liver histopathology and triglyceride content of mice. (A)** Hematoxylin–eosin or Oil Red O staining of liver sections from representative mice from each group; CV indicates lobular central vein; the original magnification is 200×. **(B)** Liver triglycerides were measured. Results are expressed per gram of tissue. Data are expressed as mean ± SD, n = 10 per group, ***P < 0.001 *vs.* control; ^*#*^ *P* < 0.05 *vs.* model.

The MCD-induced NASH model was established after the mice were fed with MCD diet for six weeks. Figure 2A shows that the liver sections of the model mice stained with HE and Oil Red O displayed abundant accumulation of fat droplets in hepatocytes, together with scattered inflammatory cell infiltration and hepatocellular ballooning degeneration, which were all improved in mice treated with SNN. The steatosis grade of the liver tissue was higher in the model group than in the control group (*P* <0.001) but was significantly downregulated in the SNN group (*P* <0.05) (Table 2). The increased liver TG content (*P* <0.001), which was another proof of lipid deposition in the livers of model mice, was consistently reduced by SNN (*P* <0.05) (Figure 2B).

**Table 2:** **Liver histology evaluated by NAFLD Activity score**

**Group**	**Control**	**Model**	**SNN**
Steatosis grade	0 ± 0	2.6 ± 0.52***	0.75 ± 0.71^#^
Lobular inflammatory grade	0 ± 0	2.0 ± 0.76***	0. 5 ± 0.53^#^
Hepatocellular balooning	0 ± 0	1.4 ± 0.92**	0. 6 ± 0.52
NAS	0 ± 0	4.8 ± 1.90***	1.9 ± 0.79^#^

Data are expressed as mean ± SD, n = 12 per group, ***P < 0.001, **P < 0.01 *vs.* control; ^*#*^ *P* < 0.05 *vs.* model.

### SNN attenuated liver injury induced by MCD diet

**Figure 3:**
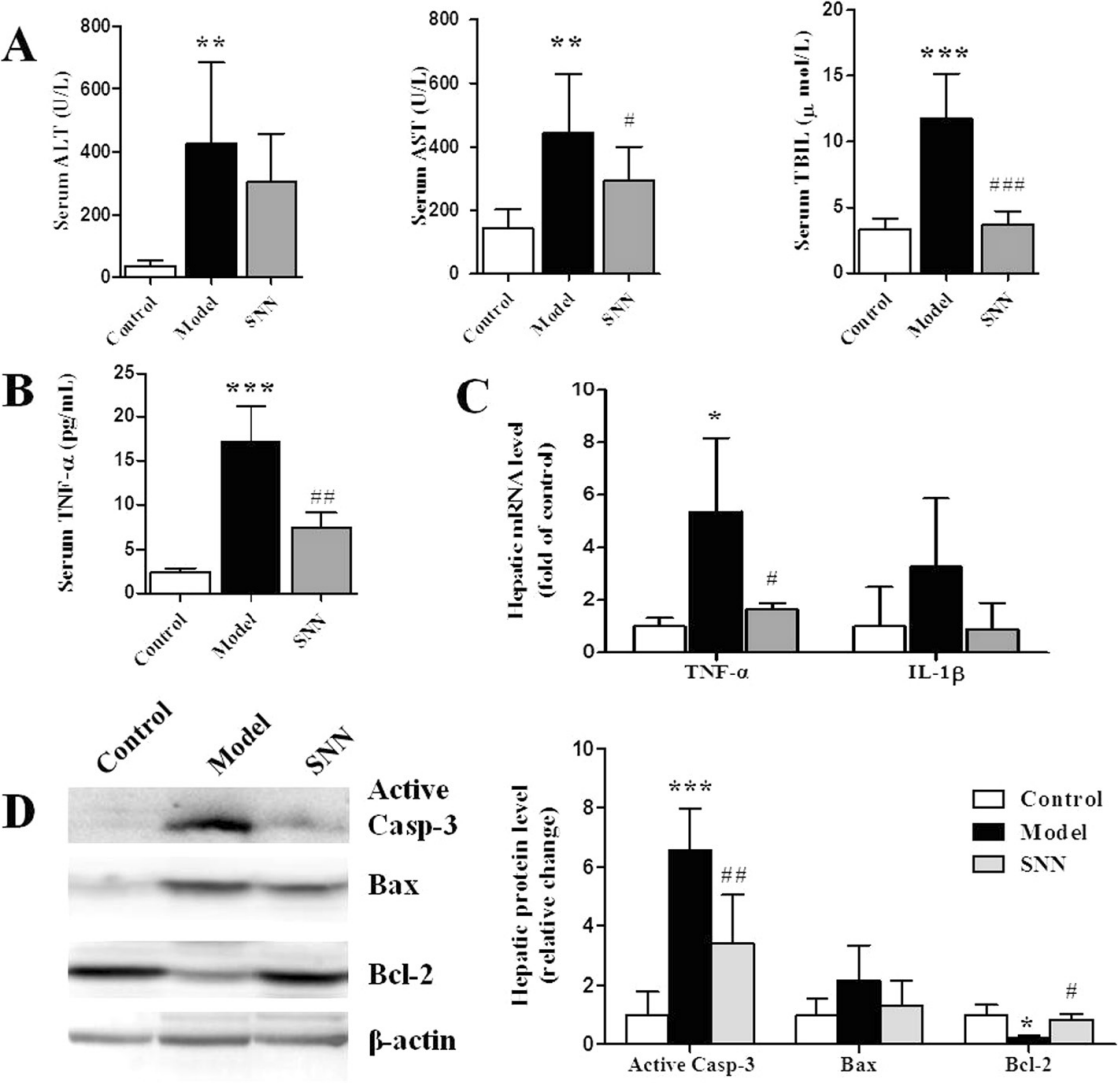
**Liver injury-related parameters of mice. (A)** Serum ALT, AST and TBIL were measured (n = 8 per group). **(B)** Serum TNF-α level was measured by Bio-Plex 200 system with Luminex system after labeling with specific Luminex magnetic beads (n = 8 per group). **(C)** Hepatic mRNA expression of the liver pro-inflammatory cytokines TNF-α and IL-1β was evaluated by quantitative real-time PCR analysis. Results were normalized to β-actin expression (n = 4 per group). **(D)** The expression of apoptosis-related genes including active caspase-3, BAX, and Bcl-2 was evaluated by immunoblot analysis of livers from four mice per group. β-actin was determined as the loading control. Data are expressed as mean ± SD, ***P < 0.001, **P < 0.01, *P < 0.05 *vs.* control; ^*###*^ *P* < 0.001, ^*##*^ *P* < 0.01, ^*#*^ *P* < 0.05 *vs.* model.

Liver damage was exhibited by the elevated serum values of ALT (*P* <0.01), AST (*P* <0.01), and TBIL (*P* <0.001) in the model group compared with the control group. SNN treatment prevented the changes to a certain extent (Figure 3A). Inflammatory cytokines were implicated in the pathogenesis of NASH. The MCD diet increased the serum level of TNF-α (*P* <0.001) and hepatic TNF-α expression (*P* <0.05) in the model group, which was blocked by SNN (Figure 3B, C). The direct evidence of liver injury was determined by lobular inflammation and hepatocyte death in liver sections. The lobular inflammatory grade (*P* <0.001), ballooning grade (*P* <0.01) and the aggregate NAS (*P* <0.001) were higher in the model mice but was downregulated in the SNN group (Table 2). The increased level of active caspase-3 (*P* <0.001) indicated more apoptosis of the hepatocytes in MCD diet-fed mice than in control mice. This phenomenon was attenuated by SNN intervention (*P* <0.01). Compared with that in the controlled mice, the anti-apoptosis molecule Bcl-2 expression was reduced (*P* <0.05) in the model mice but was upregulated in the SNN group (*P* <0.05). The pro-apoptosis molecule Bax exhibited the converse changing tendency (Figure 3D).

### SNN regulated hepatic oxidative stress in MCD diet-induced NASH

**Figure 4:**
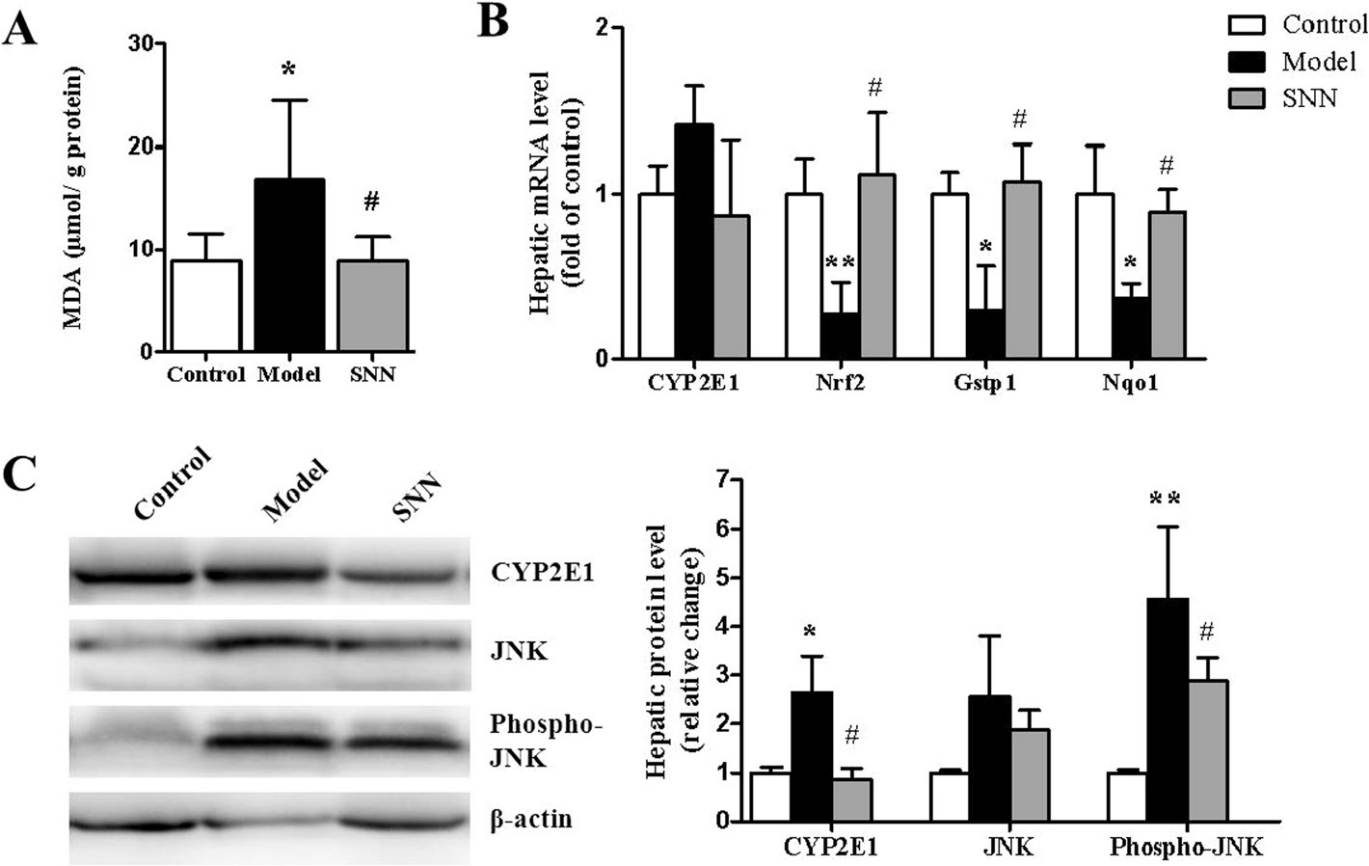
**Parameters of oxidative stress of mice. (A)** The MDA content of liver was measured. Results are expressed per gram of liver protein extract (n = 8 per group). **(B)** The hepatic mRNA expression of liver oxidant stress related genes including CYP2E1, Nrf2, Gstp1 and Nqo1 was evaluated by quantitative real-time PCR analysis. Results were normalized to β-actin expression (n = 4 per group). **(C)** The expression of CYP2E1, JNK and phospho-JNK was evaluated by immunoblot analysis of livers from four mice per group. β-actin was determined as the loading control. Data are expressed as mean ± SD, ***P < 0.001, **P < 0.01, *P < 0.05 *vs.* control; ^*#*^ *P* < 0.05 *vs.* model.

This study also found that SNN could improve the state of OS. The levels of lipid peroxidation (MDA) were significantly increased in MCD diet-fed mice compared with those in the control (*P* <0.05) but were decreased by SNN treatment (*P* <0.05) (Figure 4A). The mRNA level of CYP2E1 was not remarkably different among the groups, but its protein level was increased in the model mice and was downregulated by SNN (*P* <0.05) (Figure 4B, C). After the sixth week, the levels of nuclear factor erythroid 2-related factor 2 (Nrf2) and its target genes, Nqo1 and Gstp1, decreased in the MCD diet-induced NASH model (*P* <0.05 *vs.* control) but increased in mice administered with SNN (*P* <0.05 *vs.* model) (Figure 4B). JNK is reported as the bridge molecule of OS and apoptosis [32]. No apparent difference in JNK expression was observed among various groups. However, the level of its active form (p-JNK) increased more than fourfold (*P* <0.01) in the model mice and was reduced in the SNN group (*P* <0.05) (Figure 4C).

## Discussion

NASH progression is mediated by an inflammatory process in the liver with concomitant tissue damage. MCD diet-induced model is considered convenient for examining the role of OS and inflammation in the progression of NAFLD [33-35]. Choline and methionine are essential components for very-low-density lipoprotein (VLDL) and lipid oxidation. The deficiency of these components can reduce lipid oxidation and lipid transport outside of the hepatocytes, resulting in lipid accumulation in the liver. Choline and methionine deficiency also causes loss of active methyl which is important for glutathione cycle, leading to decreased antioxidants and promotion of OS [36,37]. The mice fed with MCD diet lost weight because of reduced caloric intake, which is in contrast to humans with NASH who are mostly obese. Thus, differences exist between MCD dietary mouse model and human NASH [37]. However, the MCD NASH model is one of the best established and commonly used dietary models for studying the evolution of inflammation and OS changes associated with NASH [38-40]. Liver injury and OS rapidly occur in MCD diet-fed model compared with other nutritional models. In particular, severe pericentral steatosis may develop in MCD diet-fed model after one week to two weeks; necro-inflammation may occur after two weeks; progressive pericellular and pericentral fibrosis may appear after eight weeks; and markedly enhanced OS can be observed from three weeks of MCD diet. Moreover, the liver injury induced by MCD diet is similar to human NASH [33,36,37]. In this study, MCD diet was used to feed C57BL/6 J mice for six weeks. Compared with the mice fed with control diet, hepatic steatosis appeared in the liver tissue accompanied with hepatocellular ballooning and scattered inflammatory cell infiltration, and transaminase activities were significantly increased in the MCD diet-fed mice, which indicate the establishment of the NASH model.

This study demonstrated the strong effect of SNN on preventing NASH development by simultaneously administering MCD diet to mice. The mice fed with MCD diet and administrated with SNN for six weeks exhibited only mild lipid deposition in their livers, instead of the prevalent hepatic steatosis, inflammation and hepatocellular ballooning degeneration that occurred in the NASH model without intervention. Consistently, the hepatocellular injury indicated by the increased serum levels of ALT, AST, bilirubin, and TNF-α and cell apoptosis in the model mice were evidently alleviated in the mice of the SNN group. Compared with our previous studies [22,25-27], the current study elucidated that SNN not only ameliorated steatosis but also improved the more serious liver injury in NASH. In addition, SNN blocked the excessive loss of body and liver weights and decreased the circulating lipid, which occurred in the NASH models. This function is similar to preventing a body from progressing into an unhealthy status.

Our previous studies identified insulin resistance, leptin resistance, and upregulated free fatty acid (FFA) synthesis as parts of the mechanisms involved in the effect of SNN [24-27]. The model mice in the present study showed lower serum levels of insulin and leptin, as well as hepatic FFA synthetases, such as SREBP-1c and stearol-CoA desaturase 1, than those of the control mice (data not shown). By contrast, obviously increased levels of MDA and CYP2E1 were observed in the livers of the NASH mice induced by MCD diet, as reported in previous studies [34,40]. Thus, the present study further focused on whether SNN could improve NASH through regulating OS.

OS is a disturbance between the pro-oxidant/antioxidant balance in favor of the former [41]. As an etiological factor in NASH [42], OS can initiate hepatocellular injury and secondary recruitment of inflammation [5,8]. Understanding the pathophysiological mechanisms of OS helps in the development of NASH treatment [43]. MDA is a lipid oxidant that is widely used as a marker of lipid oxidation. MDA increased in 90% of NASH patients, illustrating the increase of OS [42,44,45]. Generation of lipid peroxides results in subsequent damage to hepatic membranes, proteins, and DNA. CYP2E1 is also regarded as a common marker of OS. CYP2E1 can oxidize a variety of small molecule substrate. Superoxide anion, a byproduct of the CYP2E1-mediated metabolism, is a very potent ROS, which may serve as part of the second hit to advance the severity of NAFLD to NASH [46,47]. The results of this study showed increased CYP2E1 and MDA in the hepatic tissue of model mice, indicating the enhancement of OS. Nrf2 serves as a master regulator of a cellular defense system against OS. Upon exposure to ROS, Nrf2 dissociates and translocates into the nucleus, in which it binds to cis-acting antioxidant-responsive elements (ARE) and promotes the transcription of cytoprotective genes, such as NAD(P)H-quinone oxidoreductase (Nqo) and glutathione-S-transferase (Gst) [48]. Increased expression of Nrf2 has been reported in mice fed with MCD diet for two weeks as an adaptive response to elevated ROS [49]. However, in the present study, low levels of Nrf2 and its targeted genes, Nqo1 and Gstp1, were observed in the model mice. This result may be attributed to the exhaustion of compensatory mechanism for excess ROS after the mice were fed with MCD diet for six weeks. Previous study has found that livers of Nrf2 (−/−) mice on the MCD diet suffered more OS than their wild-type counterparts did [50]. The consequences of OS include lipid peroxidation in cell membranes, stellate cell activation in the liver leading to liver fibrosis, chronic inflammation, and apoptosis [35,43]. Moreover, ROS overproduction can promote hepatic insulin resistance, which further aggravates fatty liver [43]. Increased CYP2E1 activity correlates with the degree of steatosis and level of the inflammatory cytokines (e.g., TNF-α) involved in the pathology of NASH [6]. Thus, in the murine MCD diet-induced NASH model, hepatic injury develops as the decreased hepatocellular antioxidant defenses is overwhelmed by OS [43]. The mice of the SNN group demonstrated lower levels of MDA and CYP2E1 than the mice fed with MCD diet but exhibited more expressions of Nrf2, Nqo1, and Gstp1. Thus, administratering SNN helped reverse the OS imbalance. This finding may be attributed to the integrated function of the natural components contained in SNN, such as protopanaxadiol, berberine, and resveratrol. These components are antioxidants that could directly scavenge free radicals or regulate the correlated factors [21,51-53].

Apoptosis is a common mechanism of liver injury. The hepatocyte apoptosis assessed by terminal deoxynucleotidyl transferase dUTP nick end labeling (TUNEL) of liver biopsies and active caspases-3 and −7 significantly increases in patients with NASH and correlates with disease severity [54]. The mitogen-activated protein kinase JNK signaling pathway contributes to stress-induced apoptosis. Previous studies demonstrated that sustained JNK activation occurs with the development of steatohepatitis, which can be activated by overexpression of CYP2E1 in NASH. The JNK pathway may be a link between OS and apoptosis [32,55]. OS-associated JNK-mediated apoptosis requires the activation of the transcription factor c-Jun or induces apoptosis in a transcription-independent process by activating proapoptotic members of the Bcl-2 family, including Bim and Bax, or inactivating Bcl-2 and Bcl-xL [56,57]. Consistently, this study observed that the activation of caspase-3 and JNK increased in the MCD diet-induced NASH model, together with low expression of Bcl-2. Such finding implies that JNK may mediate apoptosis by regulating Bcl-2. SNN treatment significantly increased the level of activated caspase-3 and JNK and downregulated Bcl-2, suggesting the function of SNN in the regulation of the pathway of OS–JNK apoptosis.

## Conclusions

By administering SNN in the MCD diet-induced murine model, this study demonstrates that SNN has a beneficial effect on inhibiting hepatic steatosis and improving liver injury, including inflammation and hepatocyte apoptosis in NASH. The results of this study suggest the potential application of SNN in treating NAFLD with serious form of liver injury. However, these findings still require further validation by conducting clinical trials. SNN is a complex herbal medicine that may possess multiple pharmaceutical targets; however, in this study, the effect of regulating OS as well as subsequent anti-apoptosis and anti-inflammation functions were prominently observed to attenuate liver injury. Such functions contribute to explain the mechanism of SNN in modulating the pathophysiology of NASH. Furthermore, since OS and inflammation are also the major mechanism of alcoholic fatty liver disease [46,58,59], SNN might also have potential for the treatment of this common liver disease.
